# Gallic Acid-Dextran Conjugate: Green Synthesis of a Novel Antioxidant Molecule

**DOI:** 10.3390/antiox8100478

**Published:** 2019-10-12

**Authors:** Moacir Fernandes Queiroz, Diego Araujo Sabry, Guilherme Lanzi Sassaki, Hugo Alexandre Oliveira Rocha, Leandro Silva Costa

**Affiliations:** 1 Department of Biochemistry, Universidade Federal do Rio Grande do Norte, Natal 59.078-970, Rio Grande do Norte, Brazil; moacirfqn@gmail.com (M.F.Q.); popoh.diego@gmail.com (D.A.S.); 2 Department of Biochemistry and Molecular Biology, Universidade Federal do Paraná, Curitiba, Paraná 81.531-980, Brazil; sassaki@ufpr.br; 3 Department of Biology, Instituto Federal de Educação, Ciência, e Tecnologia do Rio Grande do Norte (IFRN), Rio Grande do Norte, Canguaretama 59.500-000, Rio Grande do Norte, Brazil; leandro-silva-costa@hotmail.com

**Keywords:** gallic acid-grafted dextran, *Leuconostoc* dextran, antioxidant polysaccharide

## Abstract

A novel derivative of dextran, dextran–gallic acid (Dex–Gal), obtained from simple conjugation with gallic acid, was synthesized by an efficient free radical-mediated method. To verify the synthesis of Dex–Gal, 1H-nuclear magnetic resonance (1H-NMR), Fourier transform infrared (FTIR) spectrometry, and high-performance size-exclusion chromatography (HPSEC) were employed. The results revealed the conjugation of gallic acid with the 15.5 kDa dextran from *Leuconostoc mesenteroides*. Dex–Gal had a molecular weight of 11.2 kDa, indicating that the conjugation reaction was accompanied by a minor degradation of Dex–Gal. In addition, Dex–Gal contained 36.8 ± 1.4 mg gallic acid per gram dextran. These molecules were also evaluated as antioxidants using total antioxidant capacity (TAC), reducing power, ferric chelation, and superoxide radical-scavenging assays. Both polysaccharides had no ferric chelation activity. In addition, Dex–Gal was more efficient as an antioxidant agent in TAC (13 times) and was more efficient than dextran in superoxide radical-scavenging (60 times) and reducing power (90 times) assays. These data demonstrate that Dex–Gal is a natural-compound-based antioxidant with potential applications in the pharmaceutical, cosmetic, and food industries.

## 1. Introduction

Polymers of bacterial origin that are obtained in an environmentally friendly manner are called “green polymers.” The production of these polymers is generally carried out using renewable carbon sources as raw materials [1]. These polymers have attracted interest from several sectors, including pharmaceutical, chemical, and food industries, owing to their beneficial biotechnological and pharmacological properties [2].

One of these green polymers is dextran, which is obtained from different microorganisms, such as *Lactobacillus*, *Streptococcus*, and *Leuconostoc* [3]. In general, dextran is defined as a homoglucan comprising monosaccharides linked with α-(1,6) bonds, with the branching points mainly, including the α-(1-3) bond and occasionally α-(1,4) or α-(1,2) bond [4]. The size, branching degree, and branching type in glucan vary according to the microorganism source [5].

Dextran has been gaining considerable interest owing to its ability to form viscous solutions and gels in the aqueous medium even at low concentrations, stability in a wide pH and temperature range, non-ionic nature, and good stability during various industrial processes [2]. These properties have extended the applications of dextran in industries in the form of an emulsifier, thickener, suspending agent, gelling agent, stabilizer, binder, coagulant, lubricant, and protective colloid [5]. The use of dextran to produce nanomaterials with antioxidant activities, such as colloidal complexes [6], nanoparticles [7], hydrogels [8], submicron particles [9], has also gained momentum. However, dextran only serves as a vehicle for the antioxidant compound in such molecules, owing to its low antioxidant capacity [10,11,12]. Hence, it may not contribute to the antioxidant effect of the resulting material.

One way to enhance the antioxidant potential of dextran and consequently improve the antioxidant activity of the resulting complexes is to modify these polysaccharides with the addition or removal of functional clusters. Among the several types of known polysaccharide modifications, the conjugation of phenolic compounds to polysaccharides may potentially increase their antioxidant capacities [13,14,15]. Domnina et al. [13] conjugated dextran to different sterically hindered phenols and managed to increase its antioxidant activity. More recently, the conjugation of two other antioxidant phenolic compounds, catechin [14] and quercetin [15], also led to the synthesis of dextran molecules with antioxidant activity.

Gallic acid (3,4,5-trihydroxy benzoic acid; GA) is a natural phenolic compound with low toxicity and is produced by different plants. It is found in large quantities in green tea and is easily extracted and purified from different plants, aside from being chemically synthesized. These characteristics make GA as an inexpensive product, which is widely used in food, pharmaceutical, and cosmetic industries for its ability to inhibit lipid peroxidation [16].

GA has known antioxidant [17], antitumor [18], and anti-metastatic activities [19,20]. Considering its pharmacological properties, GA conjugation with other molecules may stimulate or potentiate these activities in these molecules. Considering the structure and properties of phenyl radical, one can justify the use of GA in conjugation processes over other molecules. The presence of three hydroxyls in close positions in the benzene ring allows the release of hydrogen bonds with a low enthalpy of dissociation, thereby facilitating reactions with other molecules and improving the solubility of the molecule conjugated to GA. Furthermore, these three hydroxyls provide high reducing power to GA [21].

The benzene ring of GA upon conjugation to molecules, such as polysaccharides, may block inter- and intramolecular hydrogen bonds and prevent the formation of molecular aggregates, resulting in an increase in the solubility of the conjugated molecule. In addition, the hydrophilicity of the conjugated molecules may be increased with the use of carboxyl and hydroxyl groups of GA. Furthermore, the carboxyl group of GA makes it an easily conjugable molecule, as it may nucleophilically attack amino acids, monosaccharides, and other molecules [22].

Cúrcio [23] first reported the addition of GA to the polysaccharide, chitosan using a green method and demonstrated the subsequent increase in the antioxidant activity of chitosan; this was consistent with the reports from other groups [24,25,26]. Although this method is a cost-effective, easy-to-perform green method, it has already been used for the conjugation of catechin to dextran [14], but it has never been tested with the combination of GA and dextran. To the best of our knowledge, there is no study reporting the conjugation of GA to dextran.

Here, commercial dextran produced by bacteria of the genus *Leuconostoc* was conjugated with GA in an eco-friendly redox system to obtain dextran with better antioxidant activity and higher potential value in various industries.

## 2. Materials and Methods

### 2.1. Materials

Acetonitrile, iron (II) sulfate, sulfuric acid, and potassium ferricyanide (III) were obtained from Merck (Darmstadt, Germany), while dextran from *Leuconostoc mesenteroides*, Folin-Ciocalteu reagent, GA, ammonium molybdate, L-ascorbic acid, hydrogen peroxide, nitro blue tetrazolium (NBT), methionine, dextran standards, sodium phosphate, riboflavin, and sodium acetate were purchased from Sigma-Aldrich Co. (St. Louis, MO, USA). Other solvents and chemicals used in this study were of analytical grade.

### 2.2. Conjugation of GA to Dextran

**Figure 1:**
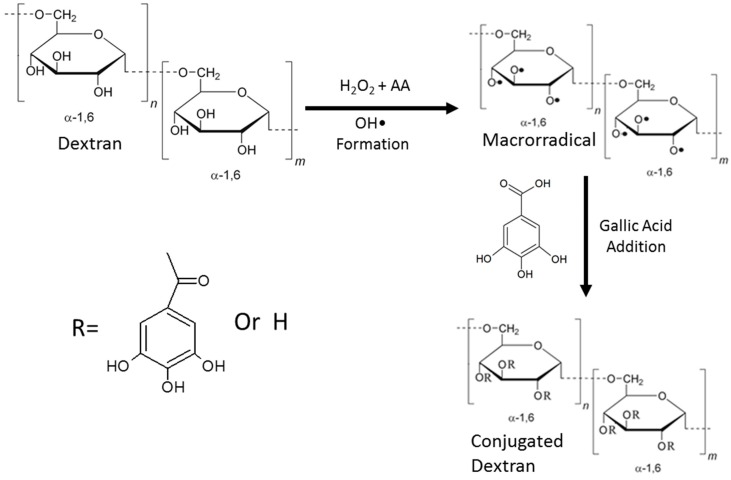
Scheme of the conjugation process of galic acid (GA) to dextran. Step 1 is the addition of the redox pair and the formation of macrorradicals. Step 2 is the addition of GA to the solution and formation of the conjugated molecule. AA—ascorbic acid; R—GA or hydrogen.

The conjugation was performed using the method proposed by Curcio et al. [23] with subtle modifications. Briefly, 500 mg of dextran was diluted in 50 mL of distilled water and mixed with 54 mg of ascorbic acid and 1 mL of hydrogen peroxide (1 M). The solution was incubated at room temperature (22 °C) for 30 min in the dark. The solution was treated with GA at a ratio of 1 mole GA to every 1 mole of dextran-repeating units (a glucose dimer was considered as a dextran-repeating unit), and the reaction was incubated at room temperature for 24 h. The solution was centrifuged using Millipore’s Amicon^®^ Ultra-centrifugal filter (Merck-Millipore, Burlington, MA, United States) with 3 kDa cut-off until all unreacted GA was withdrawn. The material was then lyophilized. In Figure 1 is showed the reaction scheme of GA conjugation to dextran.

### 2.3. Structural Analysis and Characterization of GA-dextran Conjugate

#### 2.3.1. Quantification of Phenolic Compounds

Quantification of phenolic compounds was carried out, as described by Wong-Paz et al. [27]. Dextran was used as the blank, while GA was used as the standard.

#### 2.3.2. Determination of Molecular Weight of Dextran

High-performance size-exclusion chromatography (HPSEC) (GE Healthcare Bio-Sciences, Pittsburgh, PA, USA) on TSK-Gel^®^ 3000 (30 cm × 0.75 cm) (Sigma-Aldrich, St. Louis, MO, USA) was used to determine the molecular weight of the samples. Sodium chloride (NaCl), 0.2 M in 0.05 acetate buffer was used as eluent, and the test was performed at 60 °C and 1.0 mL/min flow rate. To calibrate the column, dextran standards of 10, 47, 74, and 147 kDa were used (purchased from Sigma-Aldrich, St. Louis, MO, USA). A refractive index detector was used to detect the eluted samples.

#### 2.3.3. Fourier Transformed Infrared (FTIR) Spectroscopy

Dextran or Dex–Gal (5 mg) was mixed with potassium bromide (spectroscopic grade) and pressed to a tablet using a hydraulic press. Then this tablet was submitted to a Nexus 470 ESP FTIR spectrometer (Thermo Nicolet, Madison, WI, USA) to obtain the infrared spectra (between 500 and 4000 cm^−1^). A resolution of 4 cm^−1^ was obtained using Thirty-two scans that were evaluated and referenced against air.

#### 2.3.4. Nuclear Magnetic Resonance (NMR) Spectroscopy

Fifty milligram of dextran or Dex−Gal were dissolved in 800 μL of deuterium oxide (D2O). The 1H-NMR analysis was performed at 70 °C using a Bruker Avance III 600 MHz spectrometer (Bruker BioSpin Corporation, Billerica, MA, USA) equipped with a 5 mm QXI probe. Chemical shifts were expressed as δ values (ppm), relative to sodium trimethylsilyl propionate (TMSP) at δ = 0.00 ppm, following IUPAC recommendations.

### 2.4. Antioxidant Tests 

#### 2.4.1. Determination of Total Antioxidant Capacity (TAC)

The total antioxidant activity of the samples was evaluated by phosphomolybdenum method. This assay is based on the reduction of Mo (VI) to Mo (V) by the sample and the subsequent formation of a green phosphate complex/Mo (V) at an acidic pH [27]. Briefly, the mixture containing sodium phosphate (28 mM), sulfuric acid (0.6 M), ammonium molybdate (4 mM), and dextran samples (0.1 mg/mL) was added to a microtube, stirred, and incubated at 100 °C for 90 min. The mixture was cooled, and the absorbance measured at 695 nm wavelength. Ascorbic acid (AA) was used as standard, and the results were expressed as AA equivalent per gram of sample.

#### 2.4.2. Superoxide Radical-Scavenging Assay

As described by Presa et al. [28], 1 mL of the samples (at different concentrations) were mixture with 50 mM phosphate buffer (pH 7.8), 13 mM methionine, 2 mM riboflavin, 100 mM ethylenediaminetetraacetic acid (EDTA), 75 mM nitroblue tetrazolium (NBT) to form a 3 mL solution. This solution was kept for 10 min under light exposure at room temperature (22 °C). The entire reaction assembly was enclosed in a box covered with an aluminum foil. This reaction forms formazan that can be monitored at 560 nm. Identical tubes with distilled water and reaction mixture were used as a blank. The blanks were kept in the dark. Gallic acid was used as standard (from 0.01 to 0.6 mg/mL). The results were expressed according to the following equation:

% of activity= ([Acontrol − Asample]/[Acontrol − Ablank]) × 100

where *Acontrol*: Absorbance of the control tube, *Asample*: Absorbance of the sample tube, and *Ablank*: Absorbance of the blank tube.

#### 2.4.3. Reducing Power

One milliliter of samples in different concentrations (0.1–1 mg/mL) was mixed with phosphate buffer (0.2 M, pH 6.6) containing potassium ferricyanide (1%) and then incubated at 50 °C for 20 min. Then, 10% trichloroacetic acid (TCA) is added to the solution to stop the reaction. Ferric chloride (0.1%) in distilled water was added to the mixture, and the absorbance was measured at 700 nm, as described by Presa et al. [28]. AA is used as standard, and the results were expressed as percentage activity of 0.1 mg/mL AA, which corresponded to 100% activity.

#### 2.4.4. Iron Chelating Assay

The iron-chelating activity was determined according to the method of Melo-Silveira et al. [29]. The samples at different concentrations (from 0.1 to 2.0 mg/mL) were mixture with FeCl_2_ (2 mM). Then, ferrozine (5 mM) was added to the mixture, the solution was homogenized and incubated for 10 min at 37 °C, and the absorbance was measured at 562 nm using a microplate reader. Ultrapure water was used as blank, and EDTA is used as standard. The results are expressed in accordance with the equation:

% of chelation= ([Acontrol − Asample]/Acontrol) × 100

where *Acontrol*: Absorbance of the control tube and *Asample*: Absorbance of the sample tube.

### 2.5. Statistical Analysis

GraphPad Prism 5 (GraphPad Software Inc., La Jolla, CA, USA) was used to perform analysis of variance (ANOVA) and Student–Newman–Keuls tests (*p* < 0.05). All data are expressed as mean ± standard deviation of at least three different tests made in triplicate.

## 3. Results and Discussion

### 3.1. Characterization

#### 3.1.1. GA Dosage and Determination of Apparent Molecular Weight

The process of the conjugation of phenolic compounds using a free radical system has been frequently employed in the last few years; this includes systems that use hydrogen peroxide and ascorbic acid [14,23,26,29]. In this case, the interaction between the redox pairs of the two reactants at room temperature (22 °C) results in the formation of ascorbate and hydroxyl radicals, which initiate the conjugation reaction with GA [23]. Among the many advantages of this method, the low initiation energy allows the reaction to proceed at room temperature without the generation of any toxic waste. Moreover, the process is simple and is carried out in two steps. The first step involves activation of polysaccharide, wherein the hydroxyl radicals generated in the system interact with the polysaccharide to create macroradicals. In the second step, GA is added and gets inserted into the macroradical generated in step 1. It is important to note that this method is considered green, because it uses only water, peroxide, and ascorbate for conjugation, without the need for other solvents or reagents.

Using the aforementioned method, dextran was conjugated to GA, and the resulting molecule was called as Dex–Gal. One of the problems with this conjugation method is the requirement for a huge amount of water, owing to the need for dialysis. To avoid this waste, we used centrifugal filters (3 kDa cutoff) that are ideal for the removal of salts, sugars, nucleotides, as well as other materials with low molecular weight. Table 1 shows the GA content of the samples, as well as their molecular weight and TAC.

In comparison with the apparent molecular weight of Dex–Gal, dextran lost ~25% of its original mass after the conjugation process. To the best of our knowledge, this is the first study to demonstrate the conjugation of GA to dextran; therefore, it was not possible to compare the results of Table 1 with those of other studies. However, the application of the same GA conjugation method with chitosan resulted in the reduction in the molecular weight of chitosan between 10% [26] and 25% [29]. The interaction of the peroxide with the polysaccharide was probably responsible for generating this break in the molecule.

Despite the loss of 25% weight, GA molecules were able to maintain the antioxidant activity of the resultant GA-grafted chitosan, as reported by Wu et al. [29]. This observation was also noted with dextran (Table 1), as the native dextran molecule had no antioxidant activity in the TAC test, while Dex−GA exhibited antioxidant activity. 

No study has reported the conjugation of dextran to GA. However, the conjugation of dextran to other phenolic compounds, such as sterically hindered phenols [13], quercetin [15], and catechin [30] has already been demonstrated. Grafted dextrans exhibited better antioxidant activities than the unmodified dextran. The low solubility of quercetin and catechin demands additional steps, and non-polar solvents are required to carry out the entire conjugation process. This makes the process more expensive. However, this issue was bypassed by these authors. Consistent with the method used in the present study, these authors obtained a degree of conjugation of 19.9 mg of catechin for each 1 g of the conjugated polymer. Table 1 shows that the conjugation of 36.8 ± 1.4 mg AG/g dextran was achieved, which was equivalent to approximately 3.68% ± 0.14% (mass/mass). In other words, the value obtained by Vittorio et al. [14] was almost half the value obtained for Dex–Gal.

An important factor in GA conjugation to polysaccharides is the size of the polysaccharide molecule. Queiroz et al. [26] found that the higher the molecular weight of chitosan, the greater was the amount of AG entering the molecule. These authors suggest that this observation may be attributed to the steric interference that the entrance of each GA molecule poses to the conjugating compound. The binding of a GA molecule to the polysaccharide molecule prevents the entry of the next GA molecule. Therefore, the lower the polysaccharide amount, the better is this effect. The comparison of the data demonstrated in Table 1 with those reported by Vittorio et al. [14] highlights the proposal of Queiroz et al. [26]. The data of Vittorio et al. [14] showed the possibility of conjugating 0.068 mmol of catechin per gram of 4 kDa dextran. For Dex–Gal, about 0.208 mmol GA per gram dextran was present, indicating that about three times more GA molecules were conjugated to dextran as compared with catechin molecules. This observation points to the fact that the molecular weight of Dex–Gal is almost three times higher than that of catechin-conjugated dextran obtained by Vitorio et al. [14]. Thus, there seems a direct proportionality between the amount of phenolic compound (GA or catechin) conjugated to dextran and its molecular weight. However, more studies are warranted to prove this observation.

#### 3.1.2. FTIR Analysis

**Figure 2:**
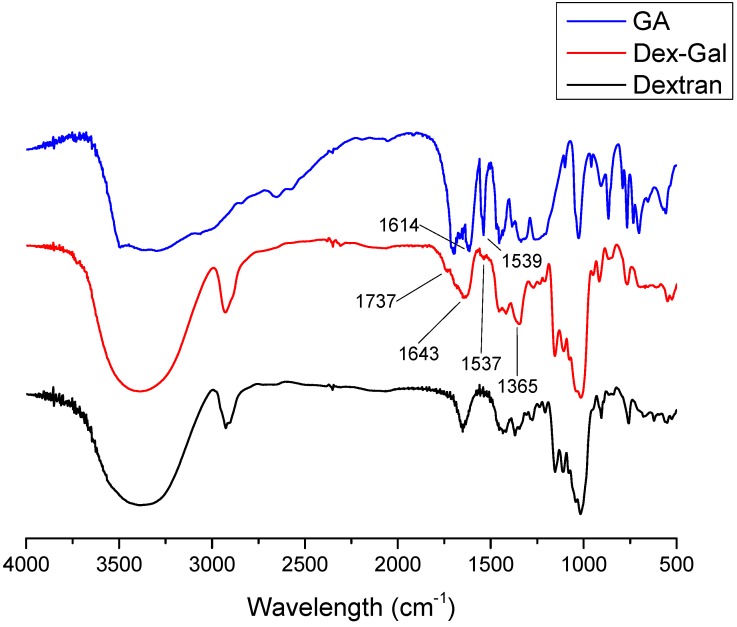
FTIR spectrum of dextran (black), GA (blue) and Dex−Gal (red).

The FTIR spectrum allows rapid identification of functional groups present in molecules. Figure 2 shows the FTIR spectra of dextran (black), Dex–Gal (red), and GA (blue). In GA spectra, the characteristic bands of this molecule could be identified; the GA spectra also included a band between 3200 and 3500 cm^−1^ representing the OH of the benzene ring. We also observed a band at 1365 cm^−1^, representative of the vibration of the OH in the plane, as well as the band at 1614 cm^−1^ corresponding to the vibration of the ring (C = C) [31], as highlighted in Figure 1.

Table 2 shows the main signals obtained from dextran and Dex–Gal spectra and their respective correlations. The characteristic bands of these molecules could be observed, including the hydroxyl vibration band around 3400 cm^−1^, the classical signal of the polysaccharides [32,33,34] (Wolkers et al. 2004, Silva et al. 2010, Melo-Silveira et al., 2012), and the band at 2900, 1421, 1153, and 1016 cm^−1^, representing C H, CO, C O C of the glycosidic bond, and the flexibility of the α-(1-6) bond, respectively, present in the dextran molecule. Moreover, we also observed bands at 906 and 850 cm^−1^ representing the pyranose ring and α-D-glucose, respectively [35,36,37].

By observing the Dex–Gal spectrum in Figure 1, it is possible to identify the similarity between this spectrum and that of the unmodified dextran. The same bands identified for dextran were detected for Dex–Gal in the form of the hydroxyl vibration band at 3400 cm^−1^ and glycosidic bond at 1153 cm^−1^ (Table 2). In addition, we also observed three bands per modification that were indicative of the binding of GA to dextran, as highlighted in Figure 1 and Table 2. The band at 1537 cm^−1^ represented the vibration of the C C bond of the aromatic ring [23], and the band observed at 1643 cm^−1^ indicated the C=C bond of the aromatic ring [31]. The most significant was the appearance of a band at 1737 cm^−1^ that was absent in both GA and unmodified dextran. This band represents the vibration of C=O as an ester bond, which indicates the binding of GA to dextran [23,31].

#### 3.1.3. NMR Analyses

**Figure 3:**
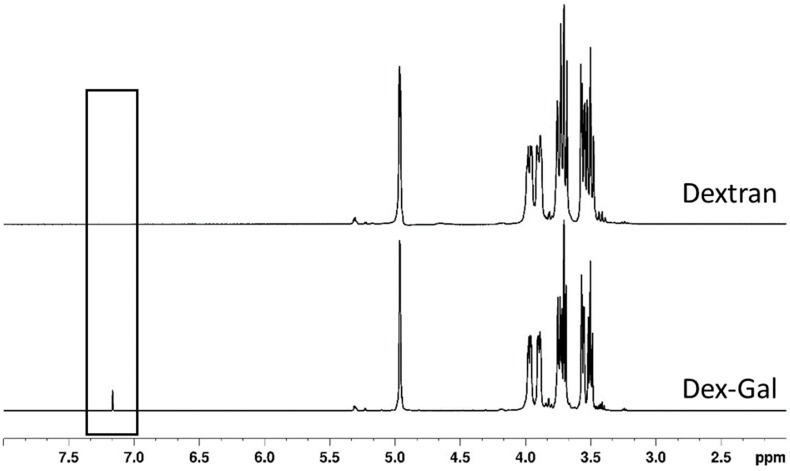
Dextran and Dex–Gal ^1^H-NMR spectra. The signal at 7.16 ppm is highlighted.

Figure 3 shows the ¹H-NMR spectra of dextran and Dex–Gal. In general, the spectra of both samples presented similar signals, consistent with the previously reported results. The signal for H-4 can be observed at 3.47 ppm, while the signals for H-2 and H-3 were detected at 3.56 and 3.69 ppm, respectively. The signals at 3.74 and 3.95 ppm corresponded to those of H6A/H6B, while the signal at 3.88 ppm belonged to H-5. The signal at 4.95 ppm was related to H1 of the α-(1-6)-linked glucose residues of the backbone. In addition, a weak signal in the region close to 5.30 ppm was observed and indicated the presence of an α-(1-3) linkage [37]. Only Dex–Gal spectrum exhibited a signal in the region of 7.16 ppm that has been already identified in GA-conjugated molecules and is associated with the H of the aromatic ring bound to the dextran chain [22,24,25].

### 3.2. Antioxidant Activities

Polyphenols form an important class of naturally occurring antioxidants, having innumerable biological activities. Among various polyphenols, gallic acid, a naturally occurring low molecular weight triphenolic compound, has emerged as a strong antioxidant [38]. Several reports showed the addition of GA to the chitosans increase in the antioxidant activity these polysaccharides [24,25,26]. However, the combination of GA and dextran was never teste. Keeping this in mind, the antioxidant activity of Dex−Gal has been evaluated in different in vitro tests.

#### 3.2.1. Ferric Chelating Activity

Both dextran and Dex–Gal failed to exhibit ferric chelating activity under all tested conditions (from 0.1 to 2 mg/mL). This data corroborates previous data that show dextrans are not good iron chelators [10]. Although GA has iron chelation activity [38], the conjugation of dextran to GA acid did not affect the chelating activity of this polysaccharide.

#### 3.2.2. Superoxide Radical-scavenging Activity

The superoxide radical has great potential to cause damage to biological systems. This radical is also a precursor of several oxidant molecules within the biological system that is known to damage the DNA, proteins, and lipids [39].

**Figure 4:**
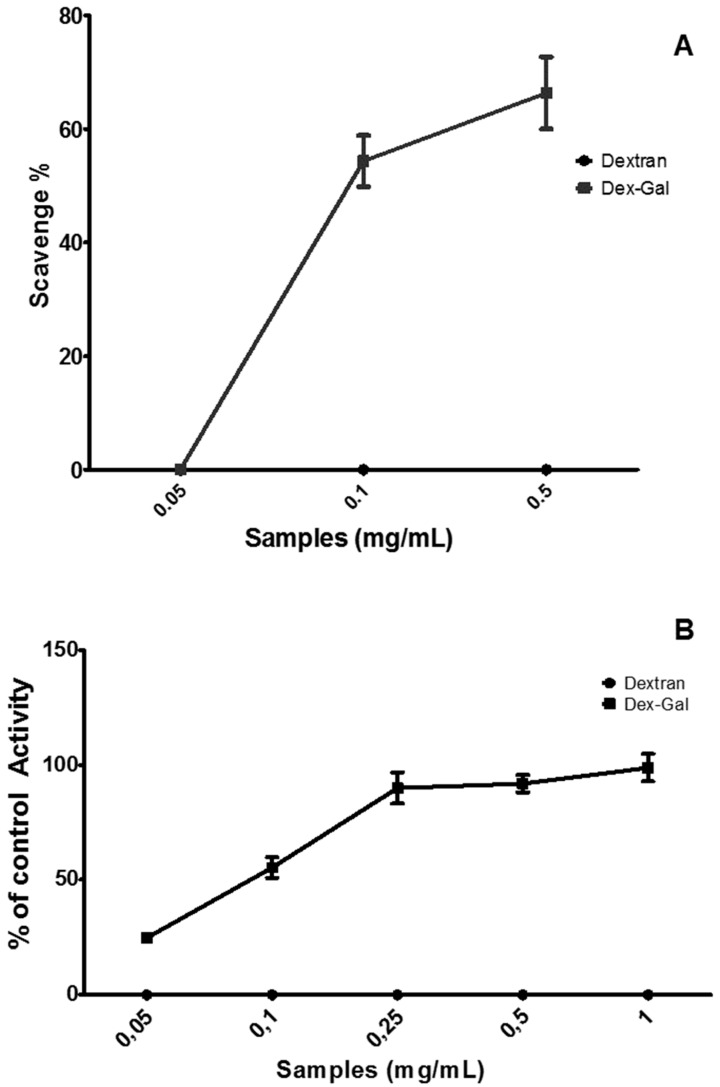
Antioxidant activities of dextran and Dex–Gal. (**A**) Superoxide scavenging activity. (**B**) Reducing power. Dextran (•) Dex–Gal (▪). The activity was analyzed with one-way ANOVA (*p* < 0.05).

Figure 4A shows the superoxide radical-scavenging activity. Dextran failed to exhibit any positive activity under the conditions evaluated. A previous study also evaluated the scavenging capacity of *L. mesenteroides* dextran superoxide ions with molecular masses of 10, 40, and 147 kDa and found that only 10 kDa dextran showed high activity (~50%); the activity did not exceed 9% for the other two types [10]. Dextrans from *Leuconostoc pseudomesenteroides* also showed low activity (about 15%) [11]. This observation is contradictory to the activity of other glucans, such as linear Β-(1,3) glucan extracted from the fruiting body of the mushroom, *Meripilus giganteus* that scavenged about 80% superoxide ions at a concentration of 0.5 mg/mL [40].

Although few articles evaluating the scavenging activity of dextran have been identified, the data show that dextran, for the most part, is not a good superoxide radical-scavenging agent. However, the association of dextran with GA modified this profile, as Dex–Gal showed a dose-dependent activity, which reached more than 60% at the maximum concentration (0.5 mg/mL). This activity comes from gallic acid. However, the amount of gallic acid (0.02 mg) present in 0.5 mg of Dex−Gal would not justify its high activity. Since GA was evaluated as superoxide radical-scavenging activity from 0.01 to 0.6 mg/mL and the maximum radical-scavengin activity value was ~80% (obtained with 0.2 mg/mL gallic acid). This value did not increase when higher concentrations were used (data not shown). These data show that the conjugation of dextran to gallic acid increased GA activity as superoxide radical-scavenging agent.

#### 3.2.3. Total Antioxidant Capacity (TAC)

The TACs of Dex and Dex–Gal are shown in Table 1. Dextran exhibited no antioxidant activity. Three other dextrans (10, 40, and 147 kDa) from *L. mesenteroides* were evaluated for this property and were shown to exert low activity, ranging from 8 to 9 equivalents of ascorbic acid per gram dextran [10].

Dex–Gal presented higher activity than *L. mesenteroides* dextran, indicating that the conjugation of dextran to GA failed to abolish the electron-donating ability of GA. Queiroz et al. [26] found that the conjugation of chitosan to GA (10 mg GA/g chitosan) resulted in a two-fold increase in its activity in the TAC test. On the other hand, Curcio et al. [23] conjugated GA with chitosan using the same method described herein and observed that although the conjugation process was successful (7 mg GA/g chitosan), the presence of GA did not increase the TAC value of chitosan. This observation shows that the conjugation of GA to the polysaccharide is not the only factor responsible for the increase in its antioxidant activity. Consistent with other types of chemical modifications, such as sulfation, the number and distribution of GA molecules, along the polysaccharide molecule, serve as important factors for improving the polysaccharide activity. Identifying these characteristics is beyond the scope of this paper. Future studies are warranted to evaluate these characteristics to determine the correlation between the structure and antioxidant activity of Dex–Gal.

#### 3.2.4. Reducing Power

The reducing power of the samples was evaluated, as described in Section 2, and the results obtained are shown in Figure 4B. Dextran had negligible activity under the conditions evaluated. On the other hand, Dex–Gal at a concentration of 0.25 mg/mL showed a reducing power of almost 100%, which remained constant with any further increase in its concentration. The reducing power of glucans, including dextran, obtained from different organisms is low [41]. For instance, laminarin (β-glucan) extracted from *Cystoseira barbata* showed 25% activity in the reducing power test at 6 mg/mL concentration [42]. Introduction of functional groups, such as sulfate and phosphate, are thought to increase the antioxidant activity of glucans, but in the case of electron donor capacity evaluated in the test of reducing power, these modifications may not greatly increase the desired activity. For instance, yeast cell wall glucans were modified to obtain sulfated glucan (S-PJ), carboxymethylated glucan (CM-PJ), phosphorylated glucan (P-PJ), carboxymethylated-phosphorylated glucan (CP-PJ), carboxymethylated-sulfated glucan (CS-PJ), and sulfated-phosphorylated glucan (SP-PJ). Evaluation of the reducing power of these glucans showed that they all had about 25% activity even at high concentrations (3.0 mg/mL) [43]. In other cases, the substitution of dextran may result in a decrease in its reducing power. For instance, the glucan obtained from the fungus *Lasiodiplodia theobromae* was subjected to different levels of carboxymethylation; and higher the degree of substitution, the lower the reducing power of the modified dextran was. The introduction of chemical groups into the polysaccharide molecules may contribute to the strengthening or weakening of the hydrogen bond dissociation energy. Therefore, hydrogen donation capacity of the polysaccharide may be increased or decreased and may consequently affect its antioxidant potential [44].

This effect does not seem to occur while evaluating the reducing power of polysaccharides conjugated with GA molecules, as the reducing power of the modified polysaccharides increased as compared to that of the original polysaccharide [24,25,26]. The hydroxyls present in the aromatic rings of the phenolic compounds are responsible for the higher increase in the reducing capacity of polysaccharides [21,45].

Dex−Gal (0.25 mg/mL) contain ~0.01 mg/mL of GA. When GA was evaluated in reducing power it showed 15% activity in this test at 0.010 mg/mL (data not shown). These data do not indicate a simple joint action of the molecules (GA and dextran) as reducing agents and that probably the conjugation of these molecules made the GA more reactive. These data corroborate the data, presented by Xie et al. [25], who combined GA and chitosan, and obtained a molecule with greater reducing power than the sum of GA and chitosan activities.

GA has a potent reducing power [25,46], as the dextran tested here showed no significant activity. One of the main objectives of the conjugation of GA to polysaccharides was to increase the bioavailability of these conjugates, as the main polyphenols in the diet may not have good bioavailability and are poorly absorbed, highly metabolized, and rapidly eliminated [47]. The effective transfer of the antioxidant properties of GA to the conjugate may improve its bioavailability and consequently enhance the antioxidant activity in the human body.

Both TAC and reducing power tests evaluate the ability of the molecule to donate electrons. This property is attributed to antioxidant compounds, because the process of formation of oxidative stress occurs in three stages, namely, initiation, propagation, and termination. Molecules that are electron donors can prevent the first step, and therefore avoid the formation of reactive species.

These two tests were used in the present study to evaluate the ability of the sample to donate electrons under different chemical conditions, consistent with the environment in living organisms, as well as during the different stages of industrial processes. If any compound manages to be an electron donor in these two tests, it exhibits a great potential to serve as an antioxidant under different conditions.

## 4. Conclusions

In this study, GA was successfully conjugated with dextran for the first time using an eco-friendly methodology with ascorbic acid/hydrogen peroxide pair as free radical initiators of the reaction. The accomplishment of this method is the absolute lack of toxic product generation. Conjugation was confirmed with ^1^H-NMR and FTIR, and the amount of GA incorporated into the polysaccharide chain was measured with the Folin-Ciocalteu reagent. The results showed that the synthesized molecule exhibited better antioxidant and superoxide-scavenging activities and reducing power than the native molecule. Further studies on the conjugation of GA to dextran should be conducted to evaluate the physical properties of dextran produced in this manner, as well as for the optimization of this method. The conjugates produced by this method may be used as dextran substitutes in various applications.

## Figures and Tables

**Table 1:** Molecular weight (MW), Gallic Acid (GA) content, and total antioxidant activity (TAC) of dextran and dextran–gallic acid (Dex–Gal).

Sample	MW (kDa)	GA Contend (mg/g)	TAC (mg/g)
Dextran	15.5	ND	ND
Dex−Gal	11.23	36.8 ± 1.4	14.8 ± 2.47

Each value represents the average of three experiments. ND stands for not detected.

**Table 2:** Main FTIR bands in dextran and Dex–Gal spectra. The bands only detected in Dex–Gal are highlighted in bold.

Band (cm^−1^)	Correlation
3400	OH vibration
2900	C–H vibration
1421	C–O vibration
1016	α-(1→6) glicosidic linkage
1153	C–O–C glicosidic linkage
906	Piranose
850	α-D-glucose
1537	C C aromatic ring
1643	C=C aromatic ring
1737	C=O ester
